# Clozapine and Solid Cancer Risk in Lithuania: A Population-Based Cohort Study

**DOI:** 10.3390/jcm15197739

**Published:** 2026-10-08

**Authors:** Mingaile Drevinskaite, Arunas Germanavicius, Giedre Smailyte

**Affiliations:** 1 Laboratory of Cancer Epidemiology, National Cancer Institute, 08406 Vilnius, Lithuania; giedre.smailyte@nvi.lt; 2 Department of Public Health, Institute of Health Sciences, Faculty of Medicine, Vilnius University, 03101 Vilnius, Lithuania; 3 Clinic of Psychiatry, Institute of Clinical Medicine, Faculty of Medicine, Vilnius University, 03101 Vilnius, Lithuania; arunas.germanavicius@rvpl.lt; 4 Republican Vilnius Psychiatric Hospital, 11205 Vilnius, Lithuania

**Keywords:** schizophrenia, clozapine, antipsychotic agents, cancer, solid tumors, cancer risk, cohort study, population-based study

## Abstract

**Background**: People with schizophrenia may experience altered cancer risk because of lifestyle-, healthcare access-, and treatment-related factors. Evidence on the solid cancer risk associated with clozapine remains limited. We aimed to assess solid cancer incidence among clozapine users in Lithuania between 2013 and 2021. **Methods**: We conducted a retrospective population-based cohort study linking National Health Insurance Fund records with the Lithuanian Cancer Registry. Patients with schizophrenia treated with clozapine during 2013–2021 were followed from the first recorded clozapine prescription until death, emigration, or 31 December 2021. Standardized incidence ratios (SIR) for cancers were calculated as the ratio of observed cancer cases in the clozapine user cohort to the expected number of cancers in the general Lithuanian population. **Results**: The cohort included 3473 clozapine users and 91 incident cancers. Overall cancer incidence was lower than expected in males. Among men with at least 3 years of clozapine exposure, prostate cancer incidence was reduced (SIR 0.47, 95% CI 0.22–0.98). Among women with at least 3 years of exposure, corpus uteri cancer incidence was increased (SIR 2.42, 95% CI 1.21–4.83). **Conclusions**: Current evidence on solid cancer incidence among clozapine users remains limited. Prolonged exposure may be associated with cancer-specific rather than uniform changes in risk. These exploratory findings should be interpreted cautiously and support continued attention to cancer surveillance and further large-scale pharmacoepidemiological studies with longer follow-up.

## 1. Introduction

Schizophrenia spectrum disorders are commonly categorized as serious mental illness (SMI), a term used for diagnosable mental, behavioral, or emotional disorders causing serious functional impairment and limiting one or more major life activities [1]. People with SMI, such as schizophrenia, are at risk of delayed diagnosis and, in some studies, increased risk of cancer-related mortality compared to the general population due to various factors, one of which could be disadvantages in the healthcare system and stigmatization [2]. Schizophrenia is a severe, chronic and often deteriorating psychiatric disorder in which relapses, cognitive impairment, social withdrawal, and loss of occupational functioning may substantially affect well-being beyond the burden of positive and negative symptoms alone [3].

Antipsychotic medications are fundamental drug therapy for treating schizophrenia. Although traditional labels such as first-generation, or typical, and second-generation, or atypical, currently available antipsychotics differ in their receptor-binding profiles and mechanisms of action [4].

The relationship between schizophrenia and cancer is frequently discussed in research; however, results are often inconclusive and can appear paradoxical [5]. Some studies showed a lower cancer incidence in patients with schizophrenia, despite higher rates of alcohol and drug consumption, smoking, lack of exercise, and poor diet, which are more prevalent in this group [6,7]. This occurrence has sparked a discussion suggesting that medications used to treat schizophrenia might have anti-carcinogenic properties [5]. In in vitro studies, some antipsychotics appear to have anti-tumour properties: pimozide and thioridazine kill breast cancer cells inhibiting cell growth. Authors have concluded that these drugs could be potential non-cytotoxic alternatives to tamoxifen for the treatment of tamoxifen-resistant human breast cancer [8]. However, a recent population-based cohort study showed a 1.26-fold higher breast cancer risk in people with schizophrenia compared to the general population. The risk of breast cancer was greater among those patients who used antipsychotic medications for one year or more than among those who used them for less than six months [9].

Clozapine is an atypical antipsychotic reserved for treatment-resistant schizophrenia because of its superior efficacy in this subgroup and is associated with lower mortality than other antipsychotics [10,11]. Its use required regular hematological monitoring because of the risk of neutropenia and agranulocytosis [11,12]. Due to its range of adverse events, clozapine is not a first-choice drug despite its beneficial effects. Recent evidence from observational studies has suggested that clozapine use may be associated with an increased risk of haematologic malignancies. A nationwide study in Finland observed that long-term use of clozapine could be associated with an elevated risk of hematological cancers, including lymphoma and leukemia [13]. Additionally, a few reports have indicated an increased risk of solid cancers among clozapine users as well.

Population-based evidence on solid cancer incidence among clozapine users remains scarce. The availability of nationwide health insurance data linked with the Lithuanian Cancer Registry provides an opportunity to evaluate cancer occurrence in a defined population of people with schizophrenia treated with clozapine. Such data may contribute to a better understanding of whether cancer incidence in this group differs from that expected in the general population.

Therefore, the aim of this study was to assess the incidence of solid cancers among people with schizophrenia who used clozapine in Lithuania between 2013 and 2021.

## 2. Materials and Methods

To assess the association between clozapine exposure and cancer risk, we conducted a retrospective cohort study in Lithuania between 2013 and 2021.

In this study, we used individual patient records from the Lithuanian Cancer registry and the National Health Insurance Fund (NHIF). Access to and linkage of national health records were authorized through the Lithuanian State Data Agency under the national health data reuse framework. The requested datasets were linked, de-identified, and provided for analysis in a secure environment accessible only to authorized researchers, in accordance with applicable data protection requirements [14]. The Lithuanian Cancer Registry is a population-based cancer registry, containing personal and demographic information (place of residence, sex, date of birth, vital status) and information on diagnosis (cancer site, date of diagnosis, method of cancer verification) and death (date of death, cause of death) of all cancer patients in Lithuania since 1978, while the NHIF gathers data on provided healthcare services, emergency and hospital admissions, and prescriptions of reimbursed medications.

In the NHIF database, we identified patients who were reported with schizophrenia (International Classification of Diseases (ICD)-10 code F20) from 2013 to 2021, the type of antipsychotic medications, and dates of prescription. Cancer cases in the study cohort were identified by linking every individual patient to the Lithuanian Cancer Registry using personal ID. Patients with any cancer diagnosis recorded before cohort entry, defined as the date of the first recorded clozapine prescription, were excluded from the study. This included patients diagnosed with cancer after the diagnosis of schizophrenia but before the first recorded clozapine prescription. Only cancer diagnoses after cohort entry were considered incident cancer events. If a patient was diagnosed with more than one primary cancer during the follow-up period, each primary cancer diagnosis was included in the site-specific analyses.

The final cohort comprised 3473 patients with schizophrenia treated with clozapine (2021 males and 1452 females).

The start of follow-up was defined as the date of the first clozapine prescription recorded in the National Health Insurance Fund database during 2013–2021. Clozapine exposure duration was defined as the elapsed time from this first recorded prescription rather than as cumulative prescription coverage. Repeated prescriptions were not summed to derive cumulative days of exposure, and gaps between prescriptions were not subtracted from the exposure duration. Because the available reimbursement data did not allow reliable identification of treatment discontinuation, patients were considered exposed from the first recorded clozapine prescription onward for the purposes of the duration analysis. Patients were categorized according to elapsed exposure duration as <3 years or ≥3 years.

Person-time was calculated from the first recorded clozapine prescription until the relevant end of follow-up, defined by death, emigration, or 31 December 2021.

Standardized incidence ratios (SIR) for cancers were calculated as the ratio of observed cancer cases in the clozapine user cohort to the expected number of cancers in the general Lithuanian population. The 46 male and 45 female observed cases include 1 male lymphoma, and the expected counts are based on registry rates for all C00–C96 sites. Corresponding population data by age, sex, and calendar year were obtained from Statistics Lithuania. Expected numbers were calculated by multiplying the person-years under observation in the cohort by sex, calendar year, and 5-year age group-specific national cancer incidence rates. The 95% confidence intervals (CI) for the SIRs were calculated using a log-normal Wald-type approximation, assuming that the observed number of cancer cases followed a Poisson distribution. Confidence limits were calculated as exp[ln(SIR) ± 1.96/√O], where O is the observed number of cancer cases.

All statistical analyses were performed using STATA 15 statistical software (StataCorp. revision 3 February 2020. Stata Statistical Software: Release 15.1. College Station, TX, USA).

## 3. Results

The study population’s baseline characteristics are described in Table 1.

Among 3473 patients with schizophrenia treated with clozapine, 91 cancer cases were observed, compared to 115.74 expected cases. Of these, 46 cancers occurred in males and 45 in females. The most frequent diagnoses of cancer in male patients were prostate cancer (11 cases, 23.9% of male cancers) and lung and trachea cancers (12 cases, 26.1% of male cancers). Among female cancer cases, the most frequent diagnoses were breast cancer (11 cases, 24.4% of female cancers) and corpus uteri cancer (9 cases, 20.0% of female cancers).

Standardized incidence ratios (SIR) are presented in Table 2. Overall cancer risk in schizophrenia patients who used clozapine was lower in males, compared to the general population SIR 0.69 (95% CI 0.52–0.92), but not in females. Males presented statistically significantly higher risk of liver cancer, SIR 3.96 (95% CI 1.65–9.51), and central nervous system cancer, SIR 3.05 (95% CI 1.14–8.13). However, it should be noted that the results for central nervous system cancer are based on less than 5 cancer cases and should therefore be interpreted with caution. It was observed that the risk of prostate cancer was lower (SIR 0.56); however, the result is borderline significant (95% CI 0.31–1.01). Among females, statistically significant elevated SIRs were observed for corpus uteri cancer, SIR 2.01 (95% CI 1.05–3.87), laryngeal cancer, SIR 11.72 (95% CI 1.65–83.21), and cancer of the eye/adnexal, SIR 9.67 (95% CI 1.36–68.67). However, the latter two estimates were each based on only one observed case and had very wide confidence intervals; therefore, they should be interpreted with considerable caution.

In further analysis, we decided to calculate the risk of the most common cancer (lung and trachea and prostate cancers for males, and breast, ovary, and corpus uteri cancers for females) in terms of the duration of clozapine use (Table 3). As mentioned earlier, the overall risk for all cancer sites combined was lower only in males; this difference was statistically significant among those who used clozapine for at least 3 years, SIR 0.49 (95% CI 0.33–0.73). We observed that in males who used clozapine for at least 3 years, the risk of developing prostate cancer was significantly lower than expected from the general population rates, SIR 0.47 (95% CI 0.22–0.98). No statistically significant difference was observed in males who used clozapine for less than 3 years. Similarly, females who used clozapine for at least 3 years had a higher risk of corpus uteri cancer, SIR 2.42 (95% CI 1.21–4.83), and no difference was observed when clozapine was used for less than 3 years. However, only 1 case was observed in this group.

Only one case of Hodgkin lymphoma was observed in a male clozapine user, SIR 0.84 (95% CI 0.12–5.99). Expected numbers for other haematologic malignancies were 2.03 for males and 1.53 for females.

## 4. Discussion

In this population-based cohort of patients with schizophrenia treated with clozapine, overall cancer incidence was lower than expected among males. In analyses according to treatment duration, prolonged clozapine exposure was associated with a lower occurrence of prostate cancer in males and a higher occurrence of corpus uteri cancer in females. These findings suggest that associations between clozapine exposure and cancer incidence may differ according to cancer site and sex; however, they should be interpreted cautiously because of the small number of site-specific cases and the observational nature of the study.

Schizophrenia affects less than 1% of the general population, and approximately 34% of patients develop treatment-resistant illness [15]. To date, clozapine remains the only approved pharmacological treatment for patients with treatment-resistant schizophrenia after inadequate response to other antipsychotics.

Despite its proven efficacy, clozapine remains underutilized today, because of mandatory blood monitoring, fear of serious adverse events, patients’ concerns about blood tests, and clinician inexperience and difficulty identifying suitable candidates for treatment [16]. Many patients receiving clozapine have complex clinical conditions and are often on polypharmacy, which can contribute to an increased burden of adverse events [17]. Globally, only a small fraction of treatment-resistant patients (about 0.2% of the adult population [18]) are prescribed clozapine.

An Australian descriptive analysis reported 384 spontaneous reports of cancers in people with schizophrenia taking clozapine between 1995 and 2020. The majority of cancer cases were hematological (104 cases), lung (50 cases), breast (37 cases), and colorectal (28 cases). They concluded that hematological cancers are the most frequent diagnosis in this group and suggested that clinicians should be aware of the possible association between schizophrenia and lymphomas. However, little attention was given to other types of cancer, even though solid tumors comprised 73.3% of cases.

In our study we observed only one case of hematological cancer among people taking clozapine, specifically, one case of Hodgkin lymphoma in males. Previous evidence from Finland indicates that the development of hematological cancers, such as those linked to clozapine, may require longer durations of treatment [13]. One reason why we observed few hematological cancer cases could be the relatively short follow-up period. Conversely, in our study, we observed 12 cases of lung and trachea cancer, and 11 cases of prostate cancer in males with schizophrenia. Among females with schizophrenia, we observed 11 cases of breast cancer.

Uwai et al. conducted pharmacovigilance analysis using the US Food and Drug Administration (FDA) adverse event reporting system database and reported 1668 cases of non-hematological cancer cases in clozapine users between 2004 and 2012 years [19]. In their analysis of adverse drug event reports, they found a 1.55% ratio of non-hematological malignant tumors among clozapine users, whereas the proportion was less than 1% for other antipsychotics (0.29% for aripiprazole and 0.32% for quetiapine and risperidone). The most prevalent neoplasm in their study was lung cancer, with 291 reported cases, and there was a notable association between clozapine and metastatic lung cancer (Odds ratio (OR) 7.48, 95% CI 5.57–10.05) or non-small-cell lung cancer (OR 2.83, 95% CI 1.79–4.46).

Although in our study we did not find statistically significant results between clozapine users and the incidence of lung cancer; nevertheless, lung cancer was the most common diagnosis. The Japanese authors suggested that monitoring lung health in patients receiving clozapine might be advisable. They also observed statistically significant results between clozapine users and the increased risk of testis cancer, OR 10.94 (95% CI 6.99–17.12), gastrointestinal carcinoma, OR 9.87 (95% CI. 7.42–13.15), throat cancer, OR 6.71 (95% CI 4.52–9.97), esophageal carcinoma, OR 5.07 (95% CI 3.69–6.95), and colon cancer, OR 1.88 (95% CI 1.43–2.47). Unfortunately, they did not report results for corpus uteri cancer, the results of which in our study were statistically significant, with an increased risk observed both overall and in a time-dependent manner.

Moreover, in our study, we observed a decreased risk of prostate cancer among clozapine users who had been on the medication for at least 3 years, a finding that aligns with Uwai et al.’s report. They observed a statistically significant decreased risk for prostate cancer, OR 0.63 (95% CI 0.42–0.95). However, the authors stressed that the study is based on a spontaneous reporting system; therefore, a reporting bias, such as over- or under- reporting, could be inevitable [19].

The mechanisms underlying the contrasting findings for prostate and corpus uteri cancer in our study cannot be determined from the present data. The lower incidence of prostate cancer may, in part, reflect differences in cancer screening, healthcare utilization, diagnostic intensity, or competing mortality among patients with schizophrenia rather than a direct protective effect of clozapine [2]. Conversely, the increased incidence of corpus uteri cancer may plausibly be influenced by metabolic factors, including obesity and glucose dysregulation, which are clinically relevant in patients receiving clozapine [12,20]. However, information on body mass index, metabolic status, lifestyle factors, and cancer-screening practices was not available in this study. Therefore, these potential explanations remain speculative and require investigation in studies with detailed information on metabolic, behavioral, and healthcare-related confounders.

In observational studies among population samples, individual cases of cancer are typically studied by including all antipsychotics, with little attention given specifically to clozapine. A Danish study examined cancer risk among neuroleptic medication users over a 14-year period. In this study, the use of neuroleptic agents was associated with a decreased risk of rectal cancer in both genders and colon cancer in female users, while some risk reduction was observed for prostate cancer (Risk Ratio 0.87, 95% CI 0.69–1.08) [21]. A Finnish study reported results of antipsychotic use and the risk of breast cancer in women with schizophrenia, with over 20 years of follow-up [22]. They found that women who used prolactin-increasing antipsychotics for five or more years had an increased risk of developing breast cancer. In contrast, the use of prolactin-sparing antipsychotics—such as clozapine, quetiapine, and aripiprazole—was not linked to an increased risk of breast cancer. The previously mentioned Danish study also observed no significant trend in the adjusted rate ratios by neither number of prescriptions or years of follow-up for breast cancer [21]. We can contribute to these findings, as we did not observe a statistically significant association between clozapine users and the risk of breast cancer.

Another population-based study observed an inverse association between antipsychotic use and gastric cancer risk [23]. Dose-dependent trends in reducing the risk of gastric cancer were shown with individual antipsychotic compounds including haloperidol, clozapine, olanzapine, quetiapine, thioridazine, amisulpride, and risperidone. The authors suggested that specific chemical structures of antipsychotics might be related to affinity and potency toward dopamine receptors, potentially affecting their antitumor effect [24]. In our study, the association between clozapine use and gastric cancer was statistically insignificant; however, our results should be interpreted with caution, as only three cases of gastric cancer were observed.

The strengths of this study include its population-based design and linkage of two nationwide data sources, allowing identification of clozapine-treated patients and cancer diagnoses within the Lithuanian population. The use of a population-based cancer registry provided systematic cancer ascertainment, while cancer-specific analyses allowed the evaluation of potential differences between individual malignancies.

Our study has several important limitations. Firstly, the possibility of reporting bias, which may underreport cancer cases among people with schizophrenia and premature mortality may reduce the opportunity for cancer diagnosis and therefore contribute to lower observed standardized incidence ratios. Secondly, the NHIF prescription and reimbursement data verify clozapine dispensing or purchase but cannot confirm actual medication usage, dose adherence or treatment interruptions. Because information on clozapine prescriptions before 2013 was unavailable, the first prescription recorded during the study period may not correspond to the actual initiation of clozapine treatment. Consequently, the total duration of clozapine exposure may have been underestimated, and analysis according to treatment duration should be interpreted with caution. Thirdly, it is important to mention that our study may be underpowered due to the relatively short follow-up period, which could lead to an underestimation of true cancer incidence, especially in cases with a prolonged cancer development. Moreover, data on confounding factors, such as smoking, alcohol and other substance use, body mass index, physical activity, diet, and socioeconomic circumstances, were unavailable to this study. Additionally, multiple cancer sites and subgroup analyses were evaluated without adjustment for multiple comparisons. Therefore, some statistically significant findings may represent chance findings due to Type 1 error, and the site-specific results should be regarded as exploratory. Finally, the study did not include a comparison group of patients with schizophrenia who were not treated with clozapine. The absence of an internal schizophrenia comparison cohort limits our ability to distinguish the effects of clozapine from those related to schizophrenia itself or other treatment characteristics.

In summary, cancer incidence among clozapine users was not uniformly altered across cancer sites. Lower overall cancer incidence was observed among males, while longer clozapine exposure was associated with lower prostate cancer incidence in males and higher corpus uteri cancer incidence in females. These findings should be considered exploratory and require confirmation in larger cohorts with longer follow-up, internal schizophrenia comparator groups, more detailed exposure assessment, and information on relevant confounding factors.

## 5. Conclusions

In this population-based cohort of patients with schizophrenia treated with clozapine, overall cancer incidence was lower than expected among males. With longer clozapine exposure, contrasting cancer-specific associations were observed, including lower prostate cancer incidence in males and higher corpus uteri cancer incidence in females. These findings suggest that cancer risk among clozapine users may vary according to cancer site rather than follow a uniform pattern. However, because of the observational design, small numbers of site-specific cases, limited follow-up, and absence of information on several important confounders, these associations should not be interpreted as causal. The findings do not currently justify changes to established cancer-screening recommendations, but they support continued attention to routine cancer surveillance in patients receiving long-term clozapine treatment. By using population-based national health data linked with the Lithuanian Cancer Registry, this study adds to the limited pharmacoepidemiological evidence concerning solid malignancies in clozapine users. Further studies should include larger cohorts, longer follow-up, internal comparison groups of patients with schizophrenia not treated with clozapine, more detailed evaluation of cumulative dose and exposure duration, and adjustment for major lifestyle, metabolic, and socioeconomic confounders.

## Figures and Tables

**Table 1:** Baseline characteristics of the study population at cohort entry (first recorded clozapine prescription.

	Male, N (%)	Female, N (%)
Number of users	2021 (58.2)	1452 (41.8)
Mean age (SD)	47.85 (16.67)	54.24 (14.95)
Duration of use		
<3 years	838 (41.5)	608 (41.9)
≥3 years	1183 (58.5)	844 (58.1)

**Table 2:** Numbers of observed (OBS) and expected (EXP) cases of cancers and standardized incidence ratios (SIR) with 95 percent confidence intervals (95% CI) in clozapine users.

				Male					Female		
Primary Cancer	ICD-10	OBS, N (%)	EXP	SIR	95% CI		OBS, N (%)	EXP	SIR	95% CI	
Stomach	C16	3 (6.5)	3.38	0.89	0.29	2.75	0 (0.0)	1.66	-	-	-
Colon	C18	1 (2.2)	2.62	0.38	0.05	2.71	2 (4.4)	2.21	0.90	0.23	3.61
Rectum, rectosigmoid, anus	C19–C21	1 (2.2)	2.38	0.42	0.06	2.99	0 (0.0)	1.56	-	-	-
Liver	C22	5 (10.9)	1.26	3.96	1.65	9.51	1 (2.2)	0.43	2.30	0.32	16.34
Pancreas	C25	1 (2.2)	1.96	0.51	0.07	3.62	2 (4.4)	1.36	1.47	0.37	5.87
Larynx	C32	0 (0.0)	1.58	-	-	-	1 (2.2)	0.09	11.72	1.65	83.21
Lung, Trachea	C33, C34	12 (26.1)	8.32	1.44	0.82	2.54	1 (2.2)	1.78	0.56	0.08	4.00
Skin, melanoma	C43	1 (2.2)	1.15	0.87	0.12	6.20	0 (0.0)	1.31	-	-	-
Skin, non-melanoma	C44	1 (2.2)	5.27	0.19	0.03	1.35	6 (13.3)	6.76	0.89	0.40	1.98
Breast	C50	1 (2.2)	0.10	10.35	1.46	73.48	11 (24.4)	11.45	0.96	0.53	1.73
Cervix uteri	C53	-	-	-	-	-	4 (8.9)	2.75	1.45	0.55	3.88
Corpus and unspecified uterus	C54, C55	-	-	-	-	-	9 (20.0)	4.47	2.01	1.05	3.87
Ovary	C56	-	-	-	-	-	5 (11.1)	2.55	1.96	0.82	4.72
Prostate	C61	11 (23.9)	19.58	0.56	0.31	1.01	-	-	-	-	-
Urinary bladder	C67	2 (4.3)	1.76	1.14	0.28	4.55	0 (0.0)	0.37	-	-	-
Eye and adnexa	C69	0 (0.0)	0.11	-	-	-	1 (2.2)	0.10	9.67	1.36	68.67
Central nervous system	C70–C72	4 (8.7)	1.31	3.05	1.14	8.13	0 (0.0)	0.87	-	-	-
Other and ill-defined sites	C76–C80	2 (4.3)	1.50	1.33	0.33	5.33	2 (4.4)	0.82	2.44	0.61	9.75
Non-Hodgkin lymphoma	C82–C85	1 (2.2)	1.18	0.84	0.12	5.99	0 (0.0)	0.93	-	-	-
All cancers	C00–C96	46 (100.0)	66.73	0.69	0.52	0.92	45 (100.0)	49.01	0.92	0.69	1.23

**Table 3:** Numbers of observed (OBS) and expected (EXP) cases of the most common cancers and standardized incidence ratios (SIR) with 95 percent confidence intervals (95% CI) in clozapine users according to duration.

			Male				
Primary Cancer	ICD-10	Duration	OBS	EXP	SIR	95% CI	
Lung. Trachea	C33, C34	<3	4	1.97	2.03	0.76	5.41
		≥3	8	6.35	1.26	0.63	2.52
Prostate	C61	<3	4	4.59	0.87	0.33	2.32
		≥3	7	14.99	0.47	0.22	0.98
All cancers	C00–C96	<3	21	15.86	1.32	0.86	2.03
		≥3	25	50.86	0.49	0.33	0.73
			**Female**				
Breast	C50	<3	5	2.95	1.69	0.71	4.07
		≥3	6	8.50	0.71	0.32	1.57
Ovary	C56	<3	3	0.66	4.53	1.46	14.05
		≥3	2	1.88	1.06	0.27	4.25
Corpus and unspecified uterus	C54, C55	<3	1	1.16	0.86	0.12	6.12
		≥3	8	3.31	2.42	1.21	4.83
All cancers	C00–C96	<3	16	12.88	1.24	0.76	2.03
		≥3	29	36.13	0.80	0.56	1.16

## Data Availability

Data is available upon reasonable request.
